# An Experimental Study on Water Permeability of Architectural Mortar Using Waste Glass as Fine Aggregate

**DOI:** 10.3390/ma13051110

**Published:** 2020-03-02

**Authors:** Guoqing Jing, Gang Huang, Wenjun Zhu

**Affiliations:** 1 School of Civil Engineering, Beijing Jiaotong University, Beijing 100044, China; 2 Shanghai Key Laboratory of Rail Infrastructure Durability and System Safety, Department of Transportation Engineering, Tongji University, Shanghai 201804, China

**Keywords:** waste glass, water permeability, metakaolin, architectural mortar, microstructure

## Abstract

This paper investigates the water permeability, consistency and density of architectural mortar with various contents of glass sand as fine aggregate. To reduce the effect of alkali-silica-reaction (ASR), metakaolin (MK) was used as supplementary cementitious material (SCM) instead of a component of white cement. The microstructure of glass sand mortar was visualized by means of scanning electron microscope (SEM) images. The experimental results showed that the permeability of the mortar increased with the glass sand, reaching its maximum at about 60–80% glass sand content. The optimum MK content varied with the content of glass sand, and higher content of MK was required for 60% glass sand. In addition, the consistency and density of mortar had a negative correlation with the increase of glass sand.

## 1. Introduction

Waste glass is one of the most important components of municipal solid waste (MSW), and has caused a heavy burden on the disposal in many cities all over the world [1]. In 2010, waste glass accounted for 4.6% of MSW in the United States [2]. In 2014, the waste glass produced by the EU reached 18.5 million tons [3]. In 2010, the waste glass produced in Hong Kong was over 370 tons per day, but only 3.3% was recycled and the rest was landfilled [1]. In 2018, the total amount of waste glass in China was about 18.8 million tons, including 9 million tons of flat glass, accounting for 48.9% [4]. On the one hand, the compositions and melting points of different glasses vary significantly, which results in the difficulty for remelting of the mixtures. The main obstacle to recycling was to separate different types of glass [5], as the discarding of waste glass is cheaper [6]. On the other hand, glass is non-degradable when used in landfills, so glass landfill is not a fundamental solution for waste glass. One possible method for the recycling of waste glass is to use it as building materials, as over 70% of the glass is SiO_2_ [5].

According to the particle size of broken waste glass, there are generally two recycling applications: glass powder as supplementary cementitious material (SCM) to replace cement, and glass cullet to replace natural aggregates in concrete or mortar [7]. A great deal of research has been done on the properties of concrete and mortar with waste glass. A considerable number of studies have focused on the alkali-silica reaction during cement hydration [7,8]. For instance, in concrete with 100% waste glass aggregate, ground granulated blast furnace slag (GGBS) can be used to partly replace white cement to reduce the alkali-silica reaction and improve the performance of concrete, including the working performance, flexural strength at 28 days, dry shrinkage, ASR risk and acid resistance of concrete [9].

Compared to metakaolin (MK), silica fume (SF), fly ash (FA) and palm oil fuel ash (POFA), glass powder has similar properties to SCM in glass aggregate concrete [8,10,11], but the bending strength, acid resistance, and mechanical performance after heating at 800 °C are even greater. It is possible to produce mortar with better performance by replacing part of cement with glass powder [12,13]. To develop the application of waste glass with different particle sizes, a series of experimental studies using glass cullet and powder in mortar were conducted by Lu et al. [14,15]. It was found that fine glass powder was able to suppress the ASR expansion caused by waste glass aggregate and enhance the strength obviously due to the pozzolanic effect and the ability to fill the microstructure.

The effects of different types of waste glass as fine aggregate on the properties of mortar or concrete have also been investigated recently. Tan et al. [16,17] studied the effects of different colors on the freshness, mechanical and durability properties and alkali-silica reactions of mortars and proposed that the mechanical properties and flowability were reduced for the glass sand as fine aggregate, but the resistance to chloride ion penetration increased. The ASR expansion was promoted for the clear glass sand, but was reduced with the green and brown glass sand. The color separation of waste glass was still considered to be one of the technical challenges to the recycling of waste glass, so waste glass with various colors is more common [18]. Special types of glass have also been studied. Choi et al. [19] investigated the feasibility of recycled heavyweight waste glass as fine aggregate in mortar. With the increase of glass content, the ASR expansion increased gradually due to the permitted value of fly ash or blast furnace slag. Wang [20] focused on liquid crystal display (LCD) glass sand concrete and found that the durability of concrete with 20% glass sand was the best, and was superior to that of the control concrete. Moreover, a slump loss was found in LCD glass sand concrete, which was consistent with the results of Ismail et al. [21]. The slump decreased by 23% and 33% at 10% and 20% glass sand content, respectively. In addition to the above characteristics, water infiltration in mortar or concrete can cause degradation or some aesthetic problems; thereby, the long-term performance and the service life of the structure were decreased [22,23]. However, only a few researchers have paid attention to permeability. For example, Lu et al. [24] studied the effect of recycled concrete aggregate and waste glass aggregate on permeability and found that waste glass aggregate concrete has a lower impermeability. Bisht et al. [25] tested the permeability of waste glass aggregate concrete at various substitution levels (18%, 19%, 20%, 21%, 22%, 23% and 24%). The results showed that with the increase of glass sand content, the permeability decreased. 

Moreover, as one of the important indicators of durability of cement-based materials, research on the impermeability of glass sand concrete has not been comprehensive enough. For example, the substitution percentage was not from 0% to 100%, and the reason for permeability change was not clear because of the different gradations of natural sand and glass sand. To study the influence of the waste glass as a fine aggregate on the permeability of the mortar, an experimental investigation was conducted in this paper.

## 2. Experimental Program

### 2.1. Materials

#### 2.1.1. Cement (C)

White ordinary Portland cement (P.W 32.5) was used in order to achieve the aesthetics of the mortar. The chemical compositions of the cement are presented in Table 1. The physical and mechanical properties of the cement are presented in Table 2.

#### 2.1.2. Metakaolin (MK)

MK is a kind of pozzolanic material, which usually acts as a suppressor agent to mitigate the ASR. The color is white, consistent with that of cement. The chemical compositions of MK are also included in Table 1, with an average particle size of 10 μm.

#### 2.1.3. Aggregates

**Figure 1:**
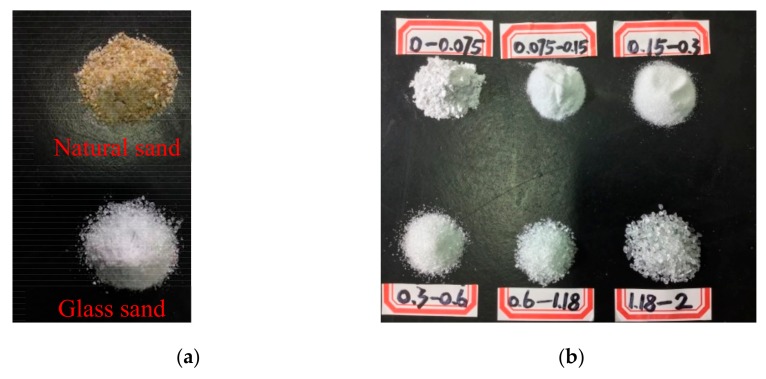
ISO standard sand and glass sand with different particle sizes. (**a**) Natural sand and glass sand; (**b**) Glass sand with different particle sizes.

**Figure 2:**
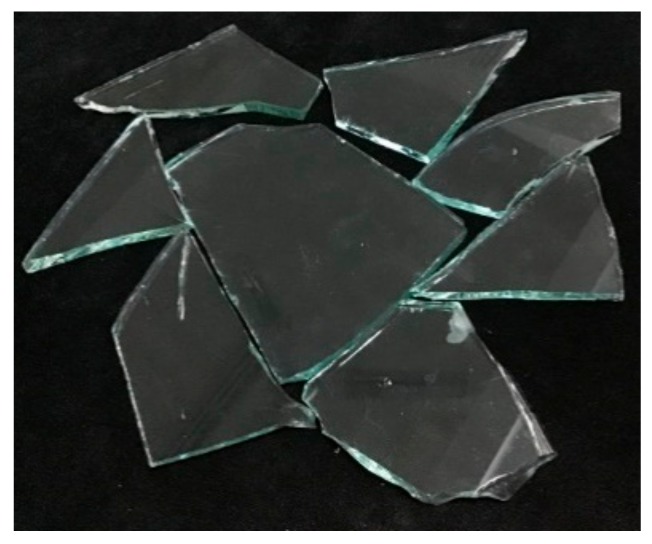
Waste flat glass.

**Figure 3:**
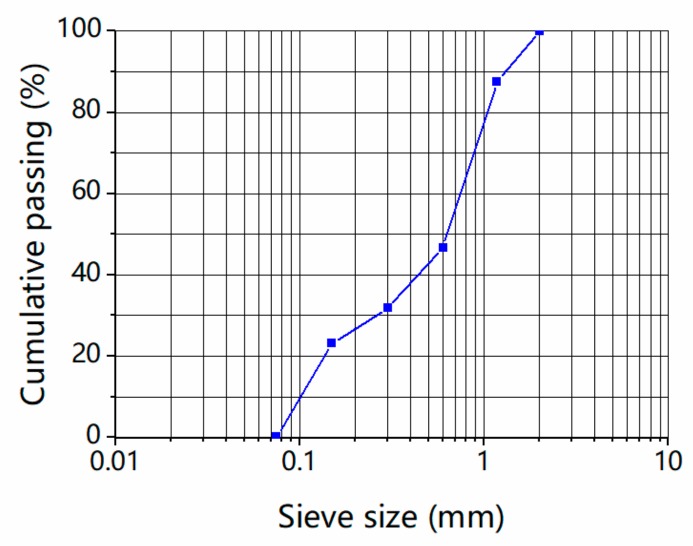
Gradation curve of aggregate.

Both natural sand with ISO standard and the glass sand are applied as fine aggregates, as shown in Figure 1. Figure 2 shows the waste flat glass in China, which accounted for 48.9% of the total amount of waste glass in China in 2018 [4]. The flat glass was crushed into particles with a size smaller than 3 mm by a hammer crusher. The particles were sieved with special gradations of 0–2 mm according to the gradation of ISO standard sand. The gradation curve of aggregate is shown in Figure 3. The fineness modulus of aggregate is 2.1.

### 2.2. Mix Proportions

The glass sand was used as a substitute for natural sand at six proportions (0%, 20%, 40%, 60%, 80%, 100%) by weight. MK accounted for 5%, 10% and 15% of the weight of cementitious materials (C + MK), respectively. The water-to-binder (W/B) ratio in this experiment was 0.4. The superplasticizer with about 0.5% of the weight of cementitious materials was adopted for the mortar to improve the workability of the mortar. Table 3 shows the details of the 20 mix proportions, including the relative weight of cementitious materials. Moreover, the figure without unit in the table is the ratio between materials.

### 2.3. Preparation of Specimens

**Figure 4:**
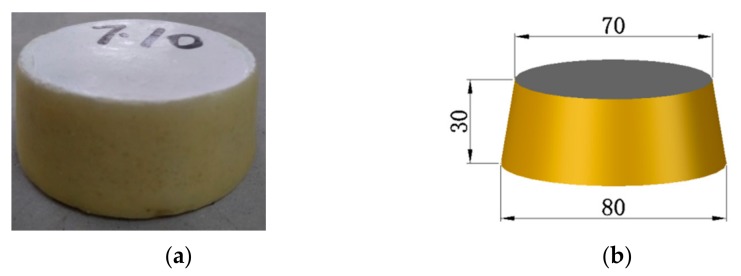
Specimen for water permeability test. (**a**) Mortar coated with sealing material; (**b**) Specimen size.

The fresh mortar paste with 20 mix proportions was cast and transferred in a room with a temperature of 20 ± 5 °C for 24 h before being demoulded. The specimens were in the shape of a truncated cone, as shown in Figure 4, with a height of 30 mm, and a diameter of the upper and lower surfaces of 70 and 80 mm, respectively, and were stored in a room with the temperature of 20 ± 2 °C and humidity over 90% for 3, 7, 14, and 28 days, respectively. A glue of paraffin rosin was applied to the side of the specimen. When the mortar paste was mixed, the water permeability test was conducted simultaneously, as well as the consistency and density tests. 

### 2.4. Experimental Methods

#### 2.4.1. Mortar Consistency Test

Mortar consistency tester is mainly composed of a cone and a container. Prior to the test, the tip of the cone (with a height of 145 mm, diameter of 75 mm, and weight of 300 ± 2 g) was placed in contact with the surface of the fresh paste in the conical container (180 mm × 150 mm). After the preparation, the fastener was opened to let the cone fall freely. When the cone had sunk in the mortar for 10 seconds, the fastener was closed and the sinking value recorded. All operations were completed within 15 min according to the Chinese national standard JGJ/T70-2009 (standard for test method of performance on building mortar [26]). The average value of the two results was taken as the consistency value. The fresh mortar with high consistency showed better workability.

#### 2.4.2. Mortar Density Test

The density test of fresh mortar was used to study the effect of different glass sand content and MK content on density. According to Chinese national standard JGJ/T70-2009 [26], a steel container (1 L) was used to load fresh mortar. After that, the container filled with fresh mortar was put on a platform vibrator for 10 s. Finally, the density was deduced by the weight of the mortar, and the average value of two repeated tests was taken. 

#### 2.4.3. Water Permeability Test

20 different mix proportions were conducted as listed in Table 3 and Table 4 at various ages: 3, 7, 14, and 28 days. Eighty groups in total and six specimens per group were evaluated based on the Chinese national standard JGJ/T70-2009 [26]. 

**Figure 5:**
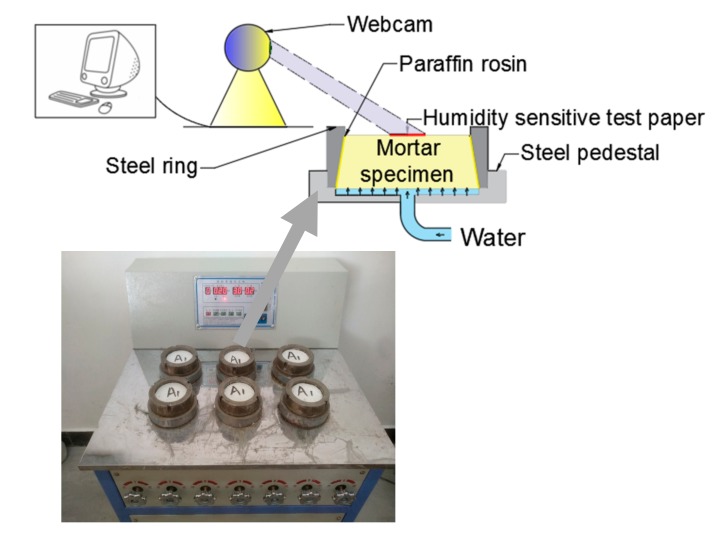
Water permeability test.

The mortar permeability tester with a capacity of six specimens per group (as shown in Figure 5) was used to investigate the water permeability of the mortar. When the six specimens were installed on the permeability tester, the machine gradually increased the water pressure to an initial value of 0.2 MPa, which was then maintained at 0.2 MPa for 2 h. According to the established program, the hydraulic pressure automatically rose by 0.1 MPa per hour until the upper surface of the third specimen became wet. The time of wetting of the third specimen was considered as the impermeability value of this group. 

**Figure 6:**
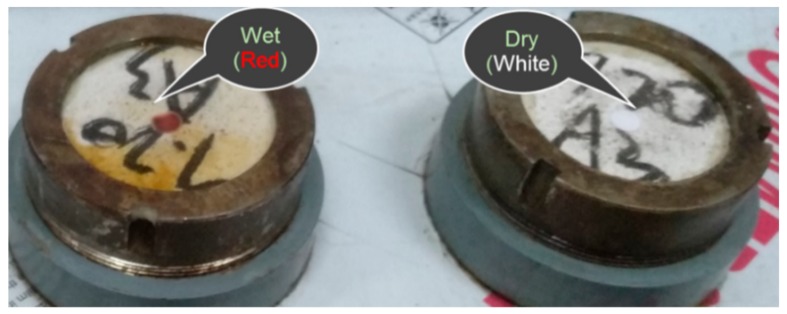
The color comparison of test paper.

An original method was proposed for this experiment during the observation. As shown in Figure 5, a humidity-sensitive test paper attached to the upper surface of the specimen was used to indicate water seepage; when the water had completely penetrated the specimen, test paper would turn from white to red (shown in Figure 6). Moreover, a webcam was adopted in this experiment to monitor the progress of the test online. As a result, the color change of the test paper was detected by the webcam, and the test data could be acquired from the computer.

## 3. Results and Discussions

### 3.1. Consistency of Mortar

**Figure 7:**
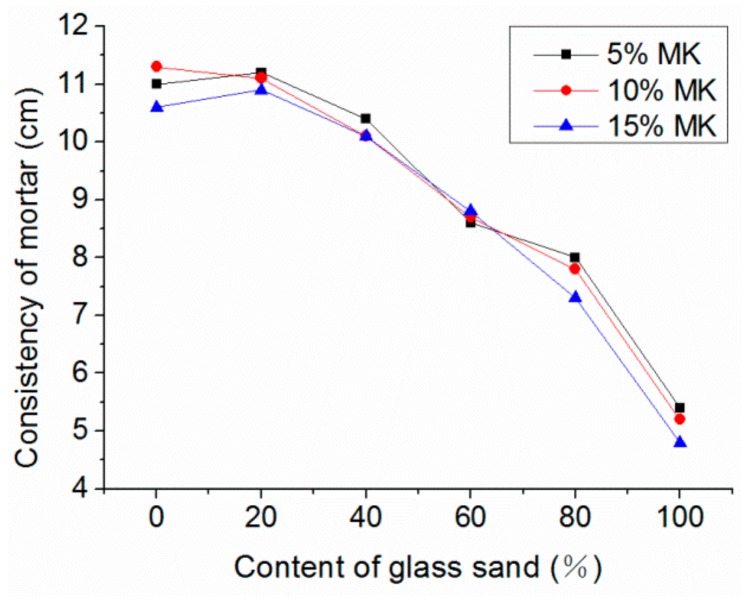
Influence of glass sand on the consistency of fresh mortar.

The consistency of fresh mortar with different percentages of glass sand and MK is shown in Figure 7. The consistency was reduced more significantly with the increase of the glass sand. Compared with the mortar without glass sand; the consistency of mortar mixed with 100% glass sand decreased by 55% at the maximum, which was similar to the fluidity and workability of the fresh mortar. Tan et al. [16] pointed out that the fluidity of the fresh mortar decreased with the increase of glass sand, which was consistent with the test results. However, Lu et al. [27] and Ling et al. [28] found that the addition of glass sand increased the fluidity of mortar. The reason for this was that the particle size of the glass sand used in the two tests was different, 0–2 mm in this experiment and a larger particle size in Lu et al.’s experiment. Compared with the natural sand, the particle size of glass sand had a higher aspect ratio and specific surface area, which increased the frictional resistance between particles and reduced the free water content in mortar.

Additionally, mortar in most groups mixed with 5% MK showed a higher consistency, while the consistency of mortar mixed with 15% MK was the lowest. Courard et al. [29] also reported that 20% MK induced a decrease of consistency of 25%, but only in ordinary mortar. When the content of the glass sand and MK were 100% and 15%, respectively, the minimum mortar consistency of 4.7 cm was reached. Moreover, the consistency decreased from 5.4 cm to 4.7 cm in the group of 100% glass sand content when MK content increased from 5% to 15%. 

### 3.2. The Fresh Density of Mortar

**Figure 8:**
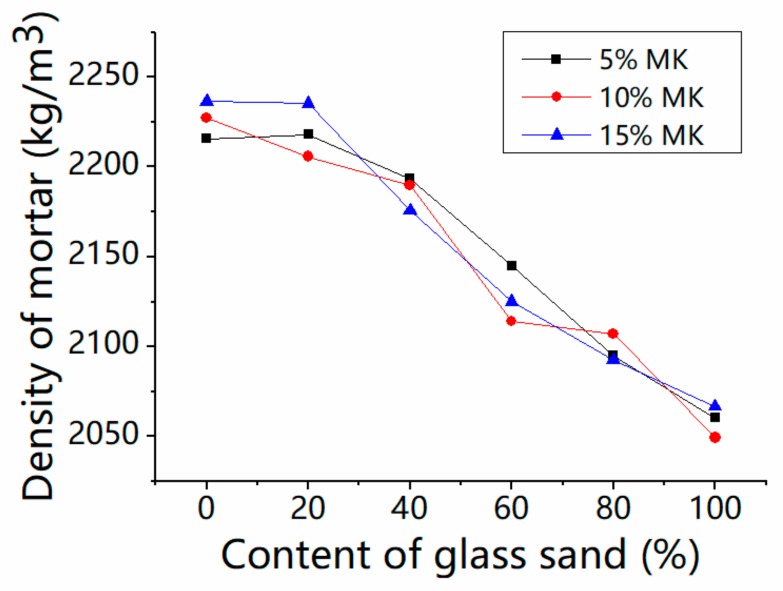
Influence on the density of the fresh mortar.

Figure 8 presents the density variation of fresh mortar with glass sand and MK. The density decreased with the addition of glass sand regardless of the different MK contents. The fresh density decreased by 2.7% and 7.9% at the maximum with the replacement percentage of 40% and 100%, respectively. Similarly, Tan et al. [16] reported that the density of mortars with different colors of glass as fine aggregates was decreased. Ismail et al. [21] also found the fresh density of concrete decreased when the glass sand was used instead of the natural fine aggregate. This was due to the angularity of glass sand [30]. The particle sizes of glass aggregates in these tests were different, but were able to reduce the density of mortar, except for special kinds of glass. From the relative position of the three curves, it can be seen that there was no obvious relationship between mortar density and MK content. The regression analysis was used to analyze the relationship between the fresh density of mortar and the percentage of glass sand. A model is proposed as follows:

Z = a × x + b(1)

where x is the percentage of glass sand, z is the fresh density of mortar, and a and b are the coefficients obtained by regression analysis. As a consequence of the analysis, a = 2242.73, b = −1.79. The correlation coefficient R^2^ of this model is 0.94, which indicates that the results of the numerical model match well with those of the real test. 

**Figure 9:**
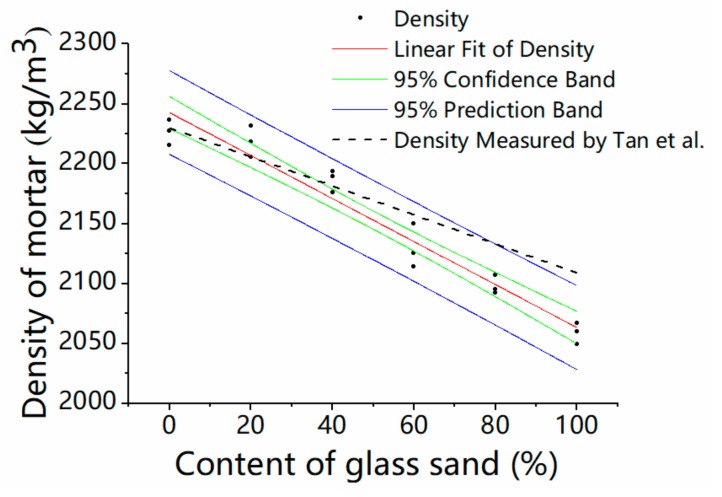
Regressed results of the fresh density.

Figure 9 shows the scatter plot of the experiment and fitting results of the regressed mode (Adj-R^2^ is 0.94, *p*-values of Intercept and x variable are 1.48 × 10^−32^ and 1.08 × 10^−11^, respectively). A reduction in density of glass sand mortar, reported by Tan et al. [16], was also included, and the results showed that the predicted model matched well with the experimental results.

**Figure 10:**
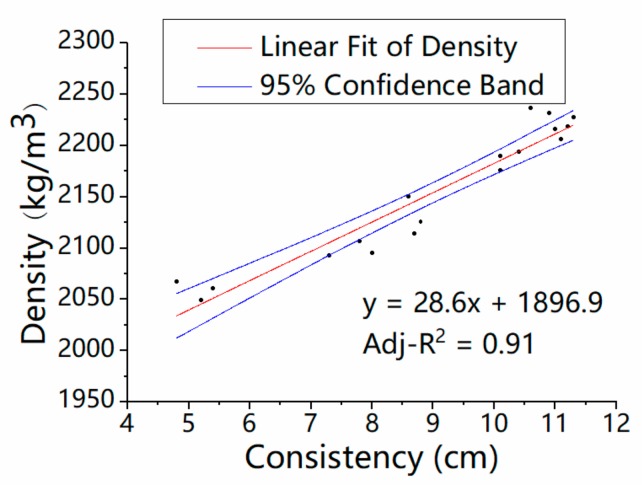
Relationship between the density and consistency of the mortar.

The consistency of mortar is one of the most important factors affecting the pore structure of mortar, which has a significant effect on the density. The density increases in a linear way with the consistency of fresh mortar, as shown in Figure 10 (*p*-values of Intercept and x variable are 2.31 × 10^−23^ and 6.32 × 10^−10^, respectively), which is similar to the fluidity of mortar. When the content of glass was changed in the range from 0% to 100%, the consistency value of the mortar increased by 50%, and the density of the mortar decreased from 2209 to 2065 kg/m^3^ in this regression model, which is in keeping with Tan’s [16] test results. Therefore, this model is also suitable for similar glass mortar.

### 3.3. Water Permeability of the Mortar

#### 3.3.1. The Relationship between Impermeability and the Content of Glass Sand with MK

**Figure 11:**
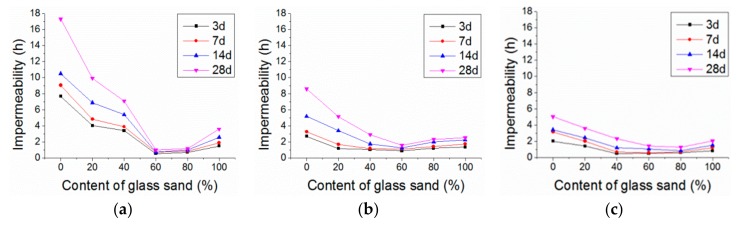
Impermeability with different content of glass sand. (**a**) 5% MK; (**b**) 10% MK; (**c**) 15% MK.

**Figure 12:**
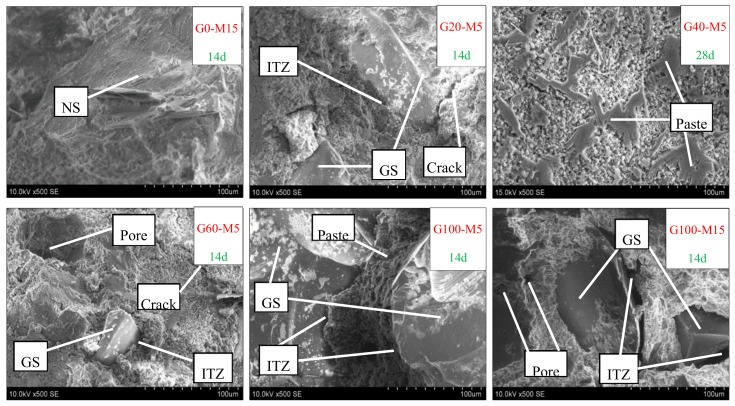
SEM images of mortar (magnification = 500, 14d: 14 days, 28d: 28 days).

Figure 11 shows the impermeability of the mortar with the glass and MK, respectively, which decreased sharply and then increased slightly with the content of the glass sand. The impermeability of the mortar with 5%, 10%, and 15% of MK at the age of 28 days reached its minimum value with glass content between 60% and 80%, and compared with 100% natural sand mortar, it was decreased by 94%, 83%, and 73%, respectively. Bisht et al. [25] reported a similar test in concrete, but the content of the glass sand only ranged from 18% to 24%. The reason for this could be the fact that there were more pores in glass sand mortar, including cracks and voids between glass and cement mortar, as the glass sand was more angular than the natural sand [25,30]. In addition, water absorption coefficient varied with the substitution level of glass sand, which characterizes the tendency of a porous material to absorb and transmit water through capillarity [31,32]. This was also supported by the results from SEM images, as shown in Figure 12. Compared with the natural sand in the group of G0-M15, the glass sand in the groups of G60-M5 and G100-M15 had a smoother surface and more edges and angles. Moreover, many cracks occurred in the interface transition zone (ITZ) of the glass sand and paste. Similar findings were found in glass particle mortar and concrete [33,34]. 

**Figure 13:**
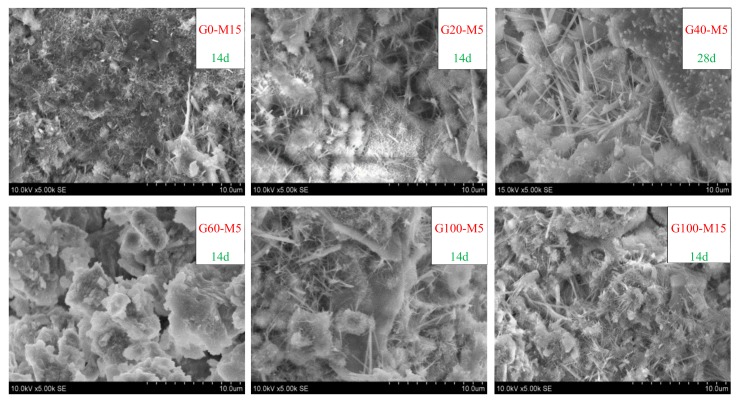
SEM images of mortar (magnification = 5000, 14d: 14days, 28d: 28 days).

Generally, the impermeability of the mortar decreased and then increased slightly, ranging from 12% to 27% in the 28d group. Penacho et al. [30] reported that the water retentivity of glass sand mortar was better because of the larger specific surface due to the cement hydration, which was supported by Neno et al. [35]. Ling et al. [28] reported that the permeable voids of the mortar increased with the content of the glass sand, but that permeable voids decreased when the content of the glass sand was high, which is directly related to the impermeability of mortar and gradually slowed down when the content of the glass sand was over 60%, and even increased with a percentage of 100%. The microstructures of mortars with different mix proportions were observed in this experiment. As shown in Figure 13, the mortar in group G60-M5 had more porous microstructures than other groups. Meanwhile, it could be found in Figure 12 that there were large pores in group G60-M5. Porous micro-structures led to lower impermeability of mortar with 60% and 80% glass sand content. 

In addition, compared with the fine aggregate used in other experiments [25,36], the size of glass sand in this experiment was smaller (0–2 mm). Glass particles with a diameter of 38–300 µm can be used as SCM. When the particle size of the glass sand was larger than 1 mm, a slight pozzolanic activity was also detected [8,37,38]. In this experiment, glass sand with a particle size no larger than 300 μm accounted for 31.8%. The characteristics of the glass sand contributed to better pore structures of mortar, especially when the content was more than 60%. On the other hand, compared with natural sand, the shape of glass sand was more irregular (as shown in Figure 12), and the particle tip of glass sand could fill large pores. Before the 60% content, there were fewer large pores, so the filling effect was not obvious. When the content of glass sand reached 60%, the large pores increased in number, and it became more obvious. Therefore, at 60%, there were the most pores and the weakest impermeability.

#### 3.3.2. Influence of MK on the Water Impermeability

**Figure 14:**
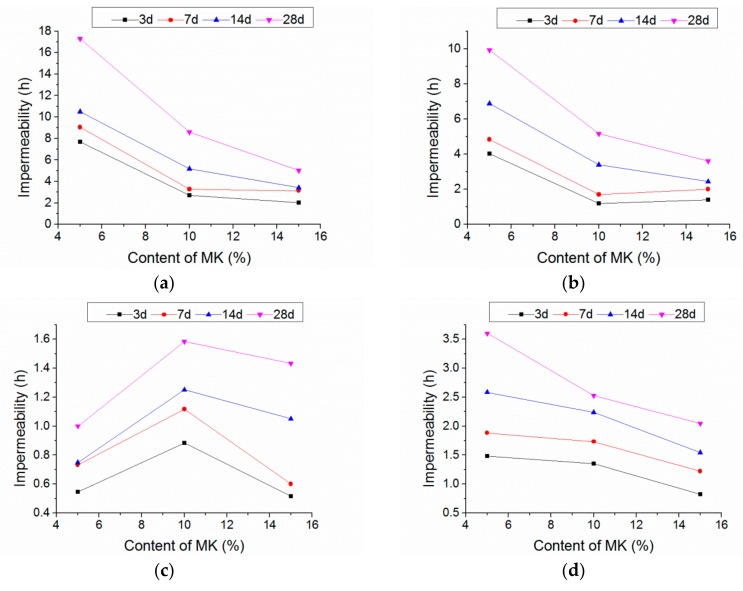
Variation in impermeability with content of MK. (**a**) Without glass sand; (**b**) 20% glass sand; (**c**) 60% glass sand; (**d**) 100% glass sand.

Figure 14 shows the impermeability of the mortars with the variation of MK and glass. When the glass content was 0%, 20%, 60%, and 100%, the optimum additions of MK were 5%, 5%, 10%, and 5% respectively. The optimum MK content increased before 60% glass sand, then decreased with the glass sand content. It was obvious that the optimum MK content varied with the glass sand content.

With the increase of the content of glass sand, the optimum MK content increased from 5% to 10%. This was caused by the “filler effect” [39] of MK as micro-aggregate. To suppress the alkali–aggregate reaction, the size of the MK particles was chosen to be only 10 μm in this test, providing the possibility for the MK particles to fill in the pores of the mortar. The pore structure of the mortar was subsequently successfully improved, along with the compactness and impermeability. However, Ling et al. [28] investigated the effect of MK on the permeable voids of the glass sand mortar, and found that the use of MK as a cementitious material increased the pore structure of the mortar. Compared with the mortar containing 10% MK, permeable voids of 20% and 30% MK mortar increased by 16% and 39%, respectively. This contradiction might be induced by the MK composition, mix proportions and content. The MK could refine the pore size in mortar [39,40]. When the content of the glass sand was low, the pore size of the mortar was small, so the improvement of MK was not obvious. However, when the content of the glass sand reached 60%, the size and the number of pores became greater [30]. As a result, proper content of MK could improve the pore structure and the impermeability of the mortar. When the glass sand content reached 100%, more cement paste was required for the larger specific surface of the glass sand. Meanwhile, the hydration of MK was slower than that of cement. Consequently, the permeability of 100% glass sand mortar decreased with the MK. 

#### 3.3.3. Regression Analysis of Impermeability

As illustrated in Figure 11, a similar curve trend can be found at different MK contents. An effective regression model was established as follows:

z = Z_0_ + B × exp{−x/C − y/D} (2)

where x is the percentage of glass sand, **y** is the percentage of MK, z is the impermeability value. Z_0_ is a coefficient related to curing age. B, C and D are the coefficients to be obtained by regression analysis as shown in Table 4. The values of adj-R^2^ were between 0.88 and 0.93.

**Figure 15:**
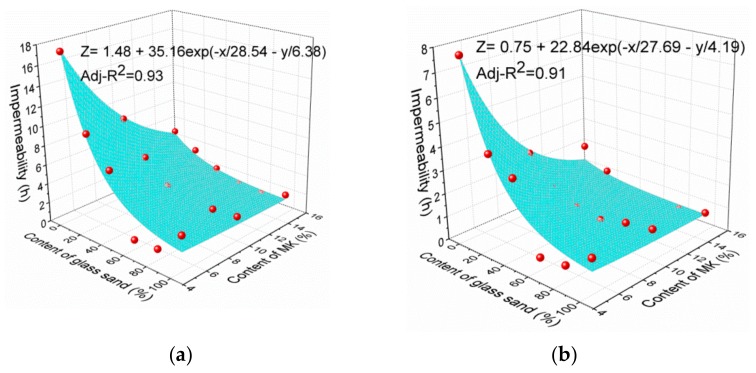
Regression models at different ages. (**a**) 28 days; (**b**) 3 days.

Figure 15 indicates the corresponding correlations between glass sand content, MK content and impermeability. This model can be used to estimate the impermeability value of similar mortar. Nevertheless, more experimental results and mix proportions are required to improve the application scope of the model.

## 4. Conclusions

In this study, the waste glass was used as a fine aggregate instead of natural sand. Meanwhile, MK was used as SCM instead of the white cement with a substitution rate of 5%, 10% and 15%, which could reduce the alkali-silica reaction. The effect of MK and glass content on the properties of mortar was analyzed. The conclusions can be drawn as follows:

1. With the increase of glass and MK content, the consistency of mortar decreased. The glass content increased the trend of the decline. The consistency of mortar mixed with 100% glass sand decreased by 55% at the maximum. The density of fresh mortar decreased by 2.7% and 7.9% with the replacement percentage of 40% and 100%. There is a regression relationship (Adj-R^2^ = 0.91) between consistency and density in order to predict the consistency.
2. The impermeability of mortar with glass sand decreased. However, it increased slightly when the glass sand reached 100%. The MK could improve the impermeability of glass sand mortar only when the glass sand content was about 60–80%.
3. SEM images showed that there was a crack between matrix and GS because of the smooth surface of glass sand, and more pore structures were found in mortar with 60% glass sand, leading to an increase of water permeability.
4. A regression model of impermeability was established that can predict the impermeability of glass sand mortar varying with glass sand content, MK content, and age.
5. As the addition of glass sand can significantly increase the permeability of mortar, glass sand mortar can be used as pervious material, and the optimum percentage is 60–80%.

## Figures and Tables

**Table 1:** Chemical compositions of cement and MK.

Chemical Composition (%)	Cement	MK
SiO_2_	15.31	52 ± 2
Al_2_O_3_	1.67	45 ± 2
Fe_2_O_3_	0.28	<0.4
CaO	63.83	<0.4
MgO	6.82	<0.2
SO_3_	2.19	-

**Table 2:** Physical and mechanical properties of cement.

Analysis	Results
Fineness	460 m^2^/kg
Normal consistency	26.7%
Initial setting time	150 min
Whiteness	90.2
Compressive strength (1d)	13.3 MPa
Compressive strength (3d)	23.5 MPa
Compressive strength (28d)	36.5 MPa

**Table 3:** Mix proportions of mortar.

Type	C	MK	ISO Sand	GS	Water	SP
G0-M0	1	0	3	0	0.4	0.005
G0-M5	0.95	0.05	3	0	0.4	0.005
G0-M10	0.90	0.10	3	0	0.4	0.005
G0-M15	0.85	0.15	3	0	0.4	0.005
G20-M5	0.95	0.05	2.4	0.6	0.4	0.005
G20-M10	0.90	0.10	2.4	0.6	0.4	0.005
G20-M15	0.85	0.15	2.4	0.6	0.4	0.005
G40-M5	0.95	0.05	1.8	1.2	0.4	0.005
G40-M10	0.90	0.10	1.8	1.2	0.4	0.005
G40-M15	0.85	0.15	1.8	1.2	0.4	0.005
G60-M5	0.95	0.05	1.2	1.8	0.4	0.005
G60-M10	0.90	0.10	1.2	1.8	0.4	0.005
G60-M15	0.85	0.15	1.2	1.8	0.4	0.005
G80-M5	0.95	0.05	0.6	2.4	0.4	0.005
G80-M10	0.90	0.10	0.6	2.4	0.4	0.005
G80-M15	0.85	0.15	0.6	2.4	0.4	0.005
G100-M0	1.00	0	0	3	0.4	0.005
G100-M5	0.95	0.05	0	3	0.4	0.005
G100-M10	0.90	0.10	0	3	0.4	0.005
G100-M15	0.85	0.15	0	3	0.4	0.005

Note: GS: glass sand; SP: superplasticizer.

**Table 4:** The result of regression analysis.

Age	Z0	B	C	D	Adj-R^2^
28d	1.48	35.16	28.54	6.38	0.93
14d	1.08	21.99	32.77	6.12	0.88
7d	0.98	22.65	26.92	4.84	0.89
3d	0.75	22.84	27.69	4.19	0.91
